# Physiology and Pathology of Diabetes

**Published:** 1888-07

**Authors:** 


THE PHYSIOLOGY AND PATHOLOGY OF DIABETES.

